# The first interactive science museum in Peru: the origin and creation of TECNO-ITINTEC, 1975-1979

**DOI:** 10.1590/S0104-59702024000100010

**Published:** 2024-04-15

**Authors:** Alejandra Ruiz-León

**Affiliations:** i Doctoral candidate, Georgia Institute of Technology. Atlanta – GA – USA Arl@gatech.edu

**Keywords:** Museum, Instituto de Investigación Tecnológica Industrial y de Normas Téncicas (ITINTEC, Science museum, History of science, Peru, Museo, Instituto de Investigación Tecnológica Industrial y de Normas Técnicas (ITINTEC, Museo de ciencia, Historia de la ciencia, Perú

## Abstract

This paper examines the development of the TECNO-ITINTEC museum, the first interactive science museum in Peru, which opened in 1979. The museum functioned under the Institute of Industrial Technology Research and Technical Standards (ITINTEC), a public institution established during the government of Velazco Alvarado. In 1975, Jorge Heraud became the president of ITINTEC’s Board of Directors and proposed a science museum to inspire future generations of scientists. José Castro Mendívil joined as the exhibition’s director and designer. Their motivation to open a museum coincided with the government’s ideals for modernization and nationalization. This article analyzes various sources including newspapers, laws that regulated the institute, reports, and interviews with people involved in the museum to understand how science and politics intersected in TECNO-ITINTEC

## Resumen

Este artículo examina la creación del museo TECNO-ITINTEC, el primer museo interactivo de ciencias del Perú, inaugurado en 1979. El museo funcionó bajo el Instituto de Investigación Tecnológica Industrial y de Normas Técnicas (ITINTEC), una institución pública creada durante el gobierno de Velazco Alvarado. En 1975, Jorge Heraud se convirtió en presidente de la Junta Directiva de ITINTEC y propuso un museo de ciencias para inspirar a las futuras generaciones de científicos. José Castro Mendívil se incorporó como director y creador de la exposición. Sus motivaciones para abrir el museo coincidieron con los ideales del gobierno militar de modernización y nacionalización. Este artículo analiza diversas fuentes incluyendo periódicos, leyes que regulaban el instituto, informes y entrevistas a personas involucradas en el museo para comprender cómo la ciencia y la política se cruzaban en TECNO-ITINTEC.

In 1979, the first interactive science museum in Peru opened its doors: TECNO-ITINTEC, as it was known, was overseen by the Institute of Industrial Technology Research and Technical Standards (the ITINTEC in its name). The institute’s stated mission was to promote industrial research and establish the technical norms that were to be used in Peru. However, the museum was not initially part of ITINTEC’s mission or functions; instead, it was the brainchild of a small group of Peruvian scientists led by the engineers Jorge Heraud and José Castro Mendívil. How did their aspirations materialize into the country’s first interactive science museum? And what political conditions paved the way to open TECNO-ITINTEC?

In this article, I argue that the museum was firmly a product of its time and place that emerged out of international science popularization trends, the Peruvian political context of the 1970s, and the relationship between these scientists and the government. Moreover, my research shows how the museum was not part of the original functions of the institute or any government education plan; rather, it was an *ad hoc* response to shifting political landscapes with a variety of intentions including inspiring the next generation of scientists. This is the first effort toward constructing the history of the TECNO-ITINTEC museum, which remains an unknown chapter in the history of science museums, the history of science in Peru, and the history of science more generally. Additionally, understanding the early years of this museum is crucial for future research on the museum’s closure in 1993, when ITINTEC was dissolved as part of the economic measures in Alberto Fujimori’s first mandate, once again beholden to political whim.

To describe the early years of TECNO-ITINTEC, I utilize various sources, including multiple materials obtained from Lima archives and interviews with the museum’s founders as well as actors involved in ITINTEC. Additionally, to understand how the museum was situated amid the politics of its origin, I analyze the laws that regulated industry during the military government. More specifically, to understand the process of opening the museum, I obtained primary sources from the National Institute for the Defense of Competition and the Protection of Intellectual Property (Instituto Nacional de Defensa de la Competencia y de la Protección de la Propiedad Intelectual, INDECOPI) archive, which holds documents related to ITINTEC and, subsequently, the museum. These documents include Castro Mendívil’s reports detailing the museum’s functions, work plan, objectives, and exhibition planning. These official sources, in turn, helped me design my survey in terms of who to interview as well as what to ask about. Finally, my research also draws from two Peruvian newspapers that covered the museum’s inauguration. Together, my analysis of these sources describes the museum’s institutional environment, the personal perspective of its founder, and the political bent of the media covering the museum.

## Historical background

The TECNO-ITINTEC museum was not born from a void. Coming into being in the early 1970s in Peru, the museum owed at least some lineage to the military government of Velazco Alvarado. But more than that, TECNO-ITINTEC was also a product of the world around it. The museum followed in the footsteps of the first interactive science museums that emerged in the late 1960s in the United States and Europe. During that time, the conflicts of the Cold War made scientific development a priority for national security in the United States and the Soviet Union (Grinell, 2003; MacDonald, 2002; Wolfe, 2003). This political interest extended to the popularization of science, which materialized in promoting a new type of science museum where the public interacted with a “friendlier” version of science that balanced education with entertainment (Bennett, 2005; Lewenstein, 1992; Oppenheimer, 1968). Such museums focused on inspiring the audience with artifacts that conveyed scientific concepts and presenting science as a modernizing activity to inspire future generations of scientists and engineers (Danilov, 1982; Kohlstedt, 2005).

This context of conflict that promoted the creation of interactive science museums also influenced their collections, which consisted of objects and hands-on activities that encouraged the public to participate through experimentation. In this sense, interactive science museums often show a sterile version of science that excludes its historical context, presenting “collections of ideas rather than things” (Semper, 2007, p.147). A common example of an object displayed in an interactive science museum is an experiment that illustrates a scientific fact but does not mention how it was discovered or any implications or controversies involved. Instead, such exhibitions “black-boxed” scientific ideas, removing them from the laborious work of “making” science (Latour, 2003). Their distinctive hands-on activities made interactive museums popular, leading to the opening of new museums of this kind worldwide.

Interactive museums signified a radical change from traditional science museums like natural history museums. They moved away from showing the historical and social context that shaped scientific knowledge to focus on the visitor’s experience and entertainment. Historians of science have criticized this decontextualized approach to science, where museums show science as a compilation of facts and concepts rather than the result of its contexts and society’s impact (Kranzberg, 1984). Even when trying to distance themselves from the context that shapes science, interactive museums were also the result of the context that influenced their creation and functioning (Ogawa, Loomis, Crain, 2009).

One of the first and most influential interactive science museums is the Exploratorium in San Francisco. This museum opened in 1969 under the leadership of physicist Frank Oppenheimer (brother of the physicist Robert Oppenheimer, involved in the Manhattan Project) and led the new interactive museums with its hands-on activities and objects designed to present scientific concepts to the general public (Hein, 1990). Museums worldwide, including TECNO-ITINTEC, replicated the Exploratorium’s approach to popularizing science; this model of promoting science through experimentation resonated with Jorge Heraud, one of the founders of the ITINTEC museum. Heraud visited the Exploratorium as a young scientist doing his PhD at Stanford. At the time, he was not yet involved with museums in Peru nor part of the Peruvian government. However, his experience at the Exploratorium traveled back with him, and many years later Heraud found a possibility to replicate the Exploratorium in Peru.

The history of TECNO-ITINTEC and other interactive science museums is part of the history of science popularization in Latin America. The region has “an especially important place in the creation of museums,” seen in their collections and role in the circulation of “people, ideas, and objects from Latin America to Europe” (Françozo, Ordoñez, 2019, p.1). Scholars of Latin America such as Podgorny and Lopes studied the natural history museums of the nineteenth century, when Latin America was a place for new institutions as well as a source for artifacts that traveled to museums around the world (Bergers, Van Trijp, 2017; Lopes, Podgorny, 2000; Podgorny, 2005; Rivera, 2011; Tagüeña, 2005). Previous works on the history of natural museums of the nineteenth century offer a starting point for studying other types of science museums. The focus shifts away from historical objects and centers on the circulated concepts and their influence on visitors.

Alongside interest in the museum collections, historians have also studied the institutional history of science museums in Latin America and the role of experts in these institutions. The creation of ITINTEC and other regional museums relied on the personal motivation of one person or a group of people for their establishment and functioning. Previous works have similarly focused on national and international experts engaged in creating spaces for science popularization such as museums, aquariums, parks, and zoos (Chastain, Lorek, 2020; Mintz, 2005; Wagensberg, 2005). Finally, on a more national level, my research adds to current efforts by Peruvian scholars to study the institutionalization of science and technology in Peru, following the work of Sagasti (Sagasti, Málaga, 2018), Cueto (2007), Marticorena (2007), Lopez Soria (2012), and many other historians of science and technology.

## The ITINTEC institute

The TECNO-ITINTEC museum was part of the ITINTEC institute, but it also had a degree of independence. The institute was a public institution intended to develop national industry while creating standardization norms in Peru. The museum shared these goals, to promote industry and scientific development. But even with the same goals, the two institutions diverged in terms of their audiences: while the museum was open to the general public, especially students, the institute was not, and instead worked directly with companies, universities, and the government to develop research projects.

The museum was, in many ways, an unintended consequence of the reformative proposals of the 1968 military government. When the museum opened in 1979, ITINTEC itself had only existed for less than a decade. The institute originated in 1968 when the Revolutionary Government of the Armed Forces led a coup d’état that ended the government of Fernando Belaunde Terry. A group of military elites, led by General Juan Francisco Velasco Alvarado, formed a new government that stressed industry and production as crucial for economic development in their “modernizing and nationalistic proposals” (Cotler, 1970, p.742). Velazco Alvarado’s government was known for its control over industry and imports.

In 1970, the military government passed a set of general laws to regulate different sectors of the Peruvian economy such as industry, mining, and fishing. General Velasco and Rear Admiral Jorge Dellepiane Ocampo, the minister of Industry, signed the new general law for industry that created ITINTEC. This law also organized different industries according to national priority and determined the tax rates for each sector (Peru, 27 July 1970, sec. V). Additionally, the law limited the importation of technology, representing “one of the highest moments of the industrialization policy by import substitution in recent Peruvian history” (Contreras, Cueto, 2007, p.370).

The primary mission of ITINTEC was to promote industrial research in Peru. The military government formally established ITINTEC in 1972 with organic law of ITINTEC DL 19565, which formalized the institute’s functions, resources, and management, which included promoting industrial-technological research, formulating research policy, evaluating companies’ research projects, and disseminating scientific and technological information (Peru, 26 Sept. 1972). Peruvian scientists and engineers interviewed for this paper remember ITINTEC as a unique environment for research and technological development unavailable in Peruvian universities during the 1970s and 1980s. But the institute’s original directives did not include opening a science museum or outreach to the community, which indicated that the museum was not part of the original plans for ITINTEC.

A unique characteristic of ITINTEC was its funding system, which has been explained in detail by Francisco Sagasti (1975) in “The ITINTEC System for Industrial Technology Policy in Peru.” The funds for ITINTEC’s research projects came directly from Peruvian companies. Each company was required to contribute two percent of its net income before taxes to industrial research. As mandated, the companies could perform their research, contract with universities, or join collaborative projects with other institutions. The role of ITINTEC was to evaluate the research proposals, approve them, and ensure their implementation.

In many cases, however, companies were either unable to or uninterested in carrying out the institute’s research projects. When this happened, the funds allocated for research went directly to ITINTEC’s bank account instead of the government’s centralized accounts. As a result, ITINTEC became a well-funded institution and “the executive agency for the formulation and implementation of industrial technology policy in Peru” (Sagasti, 1975, p.870).

Even as the institute’s services expanded through the mid-1970s to address increasingly broad audiences, museum and science popularization projects were not included in this expansion. After the establishment of ITINTEC, the government chose a group of civilian scientists to serve as the institute’s board of directors overseeing its functions and operations; the board chose Isaías Flit as the director of ITINTEC. With the August 1974 passage of the decree of law 20689 “Organic Law of the Industry and Tourism,” the institute incorporated a brand registration office and developed extension and industrial information activities (Flit, 1994). A flyer distributed in November 1975 listed ITINTEC’s three main actions: “Find, select, and obtain technical information generated by national and foreign industrial research centers. Process and store the information in a systematic way that allows easy retrieval. Disseminate information through publication and extension services targeting industry and research organizations” (Flit, Nov. 1975).

In August 1975, a second coup resulted in significant changes for ITINTEC. General Francisco Morales Bermudez coordinated a coup d’état within the Armed Forces to remove Velasco Alvarado, marking a distinct second stage for Peru’s military government. During the first stage, the country “had lost dynamism due to nationalization and the absence of private and foreign investment” (Contreras, Cueto, 2007, p.380). The new military government aimed to “redirect Peru’s economic strategy in much more conservative directions” (Hudson, Library of Congress, 1993, p.190), including hiring civilian experts to work as public servants and restore Peru’s industrial capacity. To recover this situation, General Gastón Ibáñez O’Brien assumed the post of minister of Industry and recruited a civilian to become the president of ITINTEC’s Board of Directors: Jorge Heraud.

Although initially hesitant to join the military government, Heraud accepted the position after being assured autonomy. According to Heraud (7 Nov. 2020), the minister offered him the opportunity to join ITINTEC via telephone; Heraud had returned to Peru from Stanford and worked as a researcher in the Jicamarca Radio Observatory outside Lima. Heraud initially declined and excused himself because “he was a researcher:” he was not involved with the military forces and their statist mindset. Ibáñez O’Brien assured him that the new government intended to redevelop national industry, for which they promised to give Heraud the autonomy he needed to direct the institute. Heraud saw the position as an opportunity to put his ideas into motion from within the government, so he accepted.

When Heraud joined ITINTEC, the institute approved several projects in areas that included meteorology and standardization. Heraud additionally proposed the creation of an interactive science museum to promote popular interests in science and technology and boost Peru’s industrial competitiveness. As he recalls, if the country wanted to inspire the next generation of scientists, it had to involve the people in the research process through hands-on experiences. Heraud (7 Nov. 2020) said it was “like teaching a child to read if you want to get a future Nobel Prize in Literature.”

The creation of the museum was a result of a confluence of mutually compatible circumstances including lack of opposition from the government, the institute’s autonomy over its finances and organization, and the “administrative and political capabilities of its executives” to run the institute (Sagasti, 1975, p.876). It was also very much Heraud’s idea; he recalled having “envisioned it in his mind for a while” (Heraud, 7 Nov. 2020). However, his proposal needed support from the institute’s board. In October 1975, Heraud presented the plan to the directors, who, like Heraud, had also studied in the United States; their support was immediate, since they shared his ideal to inspire future generations of Peruvian scientists by opening an interactive museum like those they had seen in the United States and Europe. To a certain extent, the government also approved the museum, or at the very least did not disapprove. The military government oversaw every aspect of society at that time, including industry, the media, and even people’s private lives (Contreras, Cueto, 2007). As a sign of its support, military members participated in the museum’s inauguration.

The museum would not have come into being without Jorge Heraud Pérez. Heraud was born in Lima in 1939 and became a distinguished Peruvian physicist. He graduated from the National University of Engineering (Universidad Nacional de Ingeniería, UNI) in 1961 with a degree in mechanical and electrical engineering; after graduation, he joined the Radio Observatory of Jicamarca (ROJ) as an intern, where he worked under the supervision of Alberto Giesecke, the director of the Geophysical Institute of Peru (Instituto Geofísico del Perú, IGP). Giesecke advised Heraud to continue his studies in the United States to pursue a career as a researcher. Heraud followed this advice and earned his master’s and doctorate in radioscience at Stanford. After graduating in 1970, he returned to Peru to continue working at the ROJ (Yánez, 2013).

Heraud was not the only scientist who followed Giesecke’s advice; 11 other young Peruvian scientists also traveled to the United States to pursue their doctorates, encouraged by Giesecke and funded by the IGP. Giesecke’s father was American, and he himself had studied in the United States (Gade, 2006). Ronald Woodman, one of the group of Peruvian scientists who studied in America, explained that Giesecke obtained the funds from a “kind of compensation, with money” that the IGP received when NASA’s Ancon station closed and was transferred to the IGP (Montoya, 2005). Giesecke expected that the Peruvian scientists would return to work at the ROJ after finishing their studies abroad. Years later, this community of Peruvian scientists supported the museum’s creation when Heraud became ITINTEC’s board president in 1975.

Heraud’s formative years at Stanford were crucial for his career as a scientist, and, according to him, also inspired the museum’s creation. As mentioned earlier, he visited the newly opened Exploratorium in San Francisco in 1969, while he was completing his doctoral work, and recognizes the influence it had on him. He remembers several visits with his wife, Cecilia Cockburn, not just on their own but occasionally guided by colleagues who collaborated in the exhibitions. Heraud saw how the museum staff designed experiments and planned the exhibits, and realized that “a science museum cannot show everything. Some things had to be hidden to stir curiosity, so the visitors come back” (Heraud, 7 Nov. 2020). The impression that the Exploratorium made on Heraud would later guide the founding ideals of TECNO-ITINTEC.

Heraud was only one half of a duo that made the museum a reality: he relied on José Castro Mendívil as director of the museum and designer of the exhibitions. Castro Mendívil was an engineer and well-known science educator in Lima. As Heraud recalls, when the board proposed the museum, they all agreed that Castro Mendívil was “the only person that could build the museum in Peru” (Heraud, 7 Nov. 2020). A contest was organized at ITINTEC to choose the museum’s director, which Castro Mendívil won easily after presenting a collection of science education objects he had created.

In July 1977, Castro Mendívil was officially hired as the museum’s director by the institute’s Board of Directors (Sesión de consejo directivo, 23 jul. 1977). At the time, he was a veteran of science popularization in Peru and a respected professor at the Pontifical Catholic University of Peru (PUCP). He incorporated hands-on experiments in his laboratory and inspired his students to promote science (Piaggio, n.d.). Castro Mendívil was also a founding member and vice-president of the Peruvian Association of Astronomy (Asociación Peruana de Astronomía, APA), for which he built the Lima Planetarium’s dome and exhibitions; the planetarium’s leadership named it after him in 1968 (Planetario José Castro Mendívil, n.d.).

The ITINTEC museum was not the first time Heraud and Castro Mendívil worked together. In an interview, Heraud recalled meeting Castro Mendívil when he was ten years old and attending events organized by the planetarium. Later, Heraud joined Castro Mendívil’s team to build the equipment for the opening of the planetarium in 1960 (Homenaje al Ing. Alberto Giesecke, conversando sobre su obra y sus pupilos, 2016; Portillo, 2018). Heraud and Castro Mendívil had many similarities: both were engineers from UNI, shared a common interest in astronomy, and believed in the importance of bringing science closer to people. Most importantly, both had traveled abroad and visited science museums, and these experiences expanded their perspectives on how to portray science to the public. Like Heraud, Castro Mendívil was inspired by interactive science museums he had visited abroad. In the early reports of the ITINTEC museum, Castro Mendívil recalled visiting the Deutsches Museum in Munich and detailed knowledge of its function.

## Creating the museum: the first steps

With the board’s approval and the hiring of the director, the ITINTEC museum entered the planning phase in 1977. The institute’s office of technology management attempted to delay the project, proposing instead that the museum open in ten years. As Heraud recalled, the board insisted that the museum project go forward. Once this obstacle was overcome, the institute’s directors projected that the museum could be built and functioning by 1981, as a report by Castro Mendívil (17 May 1977) shows.

The first step in planning the museum was to find a suitable location. The board expected construction to start in 1979, and left Castro Mendívil as the creative and technical leader of the project. He drafted architectural plans for the museum and suggested that the architects overseeing the final design make at least one trip to major science museums abroad (Castro Mendívil, 22 Mar. 1977, p.3-6). Mendívil’s insistence on visits to foreign institutions demonstrates the impact other museums had on the people behind TECNO-ITINTEC, as well as on the final museum itself. The ITINTEC architects were to learn from the characteristics of these museums, including the use of space, organization of exhibitions, and proper lighting.

In his reports, Castro Mendívil estimated that the museum would cover 3,000m^2^ and cost 60,000 million soles (approximately $2,065,769 in current US dollars). ITINTEC would provide 48 million, and donations from 20 private companies would make up the rest of the construction budget. To build the exhibits, he estimated a final cost of 15 million soles.

Although Castro Mendívil was confident in his plans, the project struggled in its first step: securing a location. The first proposal requested an area inside the Parque de las Leyendas, the Zoo of Lima. On February 8, 1977, ITINTEC officially petitioned the Lima Park Service (Servicio de Parques de Lima, SERPAR), the public institution in charge of the zoo, but SERPAR declined in an unofficial response. Instead, they suggested 3.5 hectares of land in Tupac Amaru Park, now known as La Videna, as an alternative location for the museum (Castro Mendívil, 17 May 1977, p.4). The institute’s board visited the site and issued a new request to SERPAR, which again declined on May 17, 1977.

After SERPAR’s negative responses, it was clear that the museum had to start in a temporary location while a final site was found and the building process got underway (Castro Mendívil, 20 June 1977, p.9). In June 1977, the museum’s board decided to rent a provisional space and move the museum’s opening forward to somewhere between 1977 and 1979. The directors saw the early opening as an opportunity, because “a great deal of experience will be gained in the administrative and operational aspects of a dynamic exhibition such as the one we are planning to build … that will be used when TECNO moves to its final location” (p.9).

The temporary location was a house that fit the museum’s needs, located at avenue Salaverry 2461 in the district of San Isidro; it spanned a total of 1125m^2^, including the gardens and three floors (Planos demolición acceso 15218-20200001…, n.d.). The house provided many advantages: it was centrally located in Lima, with nearby access to many bus lines, it was big enough to hold the entire exhibition, and it was divided into several rooms so that the exhibits could be organized by themes. Additionally, one room functioned as an auditorium.

The house was the property of Mrs. Esther Asín Llona de Correa, an upper-class woman who had acquired the house with her late husband, Jorge Correa Santisteban, in May of 1946. Mrs. Asín’s husband and daughter had already died when the museum rented the property (Partida de Registros Públicos N 49072025, n.d.). Mrs. Asín signed the contract with Isaías Flit, the director of ITINTEC, in 1979. The agreement was for one year at 120,000 soles per month, approximately $3,551.51 US dollars today. Later, in June of 1979, after the museum had opened, the institute renewed the contract for an additional year for 150,000.00 soles per month (US$4,439.37) (ITINTEC, 1979). The museum remained in that location until 1983, when Asín opted not to renew the contract, according to Jorge Heraud. After that, she occupied the house until 1997, when she sold it to a company that demolished it and sold the land in 2004. Today, the location is a parking lot for Cayetano Heredia University (Partida de Registros Públicos N 03024345, n.d.).

## TECNO-ITINTEC opens its doors

The process of opening TECNO-ITINTEC required many rounds of modifications of the museum’s functions, names, and exhibits. The museum’s reports reveal that the directors also had to modify their expectations for the museum to be accepted by ITINTEC’s board members. In the first report of March 22, 1977, Castro Mendívil (22 Mar. 1977, p.1) pointed out that lack of scientific knowledge and its industrial applications was “one of the surest causes of underdevelopment,” and that the museum was therefore the “safest, fastest, most efficient, and economical” (p.2) way to reach the vast majority of people to rectify this problem.

After the museum’s opening, ITINTEC included the museum guidelines as part of an official document. The document is signed not by Castro Mendívil, but by Luis Paretto Olivera (20 Aug. 1979), ITINTEC’s technical advisor on general management. The document lists four objectives for the project:

1. Encourage general interest and national inventiveness in scientific and technological activities to contribute to the industrial and socio-economic development of the country.
2. Strengthen scientific and technological training for technicians, students, and the general public to promote technical and industrial progress at the national level.
3. Promote industrial outreach and disseminate scientific and technological knowledge through the expansion and diversification of basic and applied science experiments in the industrial and artisanal fields that provide guidance and motivation to young people and adults to observe, consider, and seek technological solutions.
4. Promote the exhibition of products and processes from national industry, particularly highlighting those that use proprietary technology obtained as a result of research projects conducted by industrial companies and generated or financed by ITINTEC.

This set of objectives differs from the original ones listed by Castro Mendívil in previous reports. First, the initial goals were more aligned with education, such as influencing students, teachers, and people with scientific interests. Although the educational purpose of the museum remained, its focus shifted to national industrial development. Now, the museum’s role was more closely aligned with the promotion of ITINTEC (and, in turn, the goals of the military government), beyond just a museum looking to inspire future generations of scientists. As seen in the reports, the museum’s founders eventually reframed their goals in line with the institute so that that it could open function.

**Figure 1:**
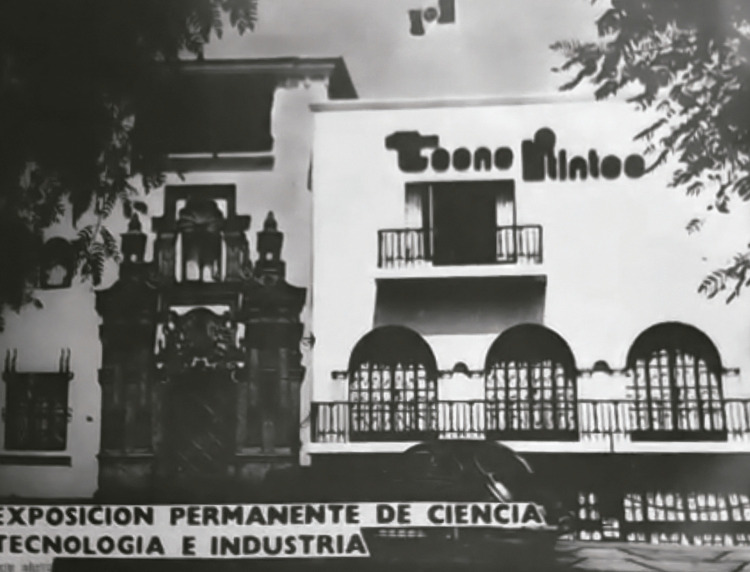
: Museum façade, showing the TECNO-ITINTEC logo but no indication that the building contained a museum open to the public (Source: Photograph courtesy of Jorge Heraud, recorded during a video call)

Before the museum opened, it went through a process of choosing a definitive name. In the late 1970s, naming a museum was not the strong marketing strategy it later became in the 1990s (Caldwell, 2000), but that did not make this process less critical. Initially, Castro Mendívil (22 Mar. 1977) presented the project as the “Permanent Exposition of Science, Industry, and Technology” (Exposición Permanente de Ciencia y Tecnología, EPCIT), but later opted for the “Museum of Science and Technology,” which excluded any reference to industry and marked a shift away from both government and ITINTEC priorities (Castro Mendívil, 17 May 1977). In June 1977, the museum settled on a name: “TECNO-ITINTEC, Permanent Exhibition of Science and Technology.” Note the absence of the word “museum.” The abbreviated TECNO-ITINTEC referred to technology as the central theme of both the museum and the host institutions; it was used for the museum’s logo, communication materials, and façade (Figure 1).

The museum’s opening date was also a point for deliberation for the founders. They considered the opening an important event for the museum’s success because it would stir interest among visitors and sponsors. ITINTEC’s directors needed to be flexible with the opening date until the experiments and exhibitions were ready for display. In one of the reports, Castro Mendívil stressed the need to promote the inauguration, including media appearances, advertising, and a hot air balloon (Castro Mendívil, 17 May 1977, p.7-8). He was partially successful: there was no balloon, but the local newspapers *El Comercio* and *Expreso* featured five stories about the museum.

**Figure 2:**
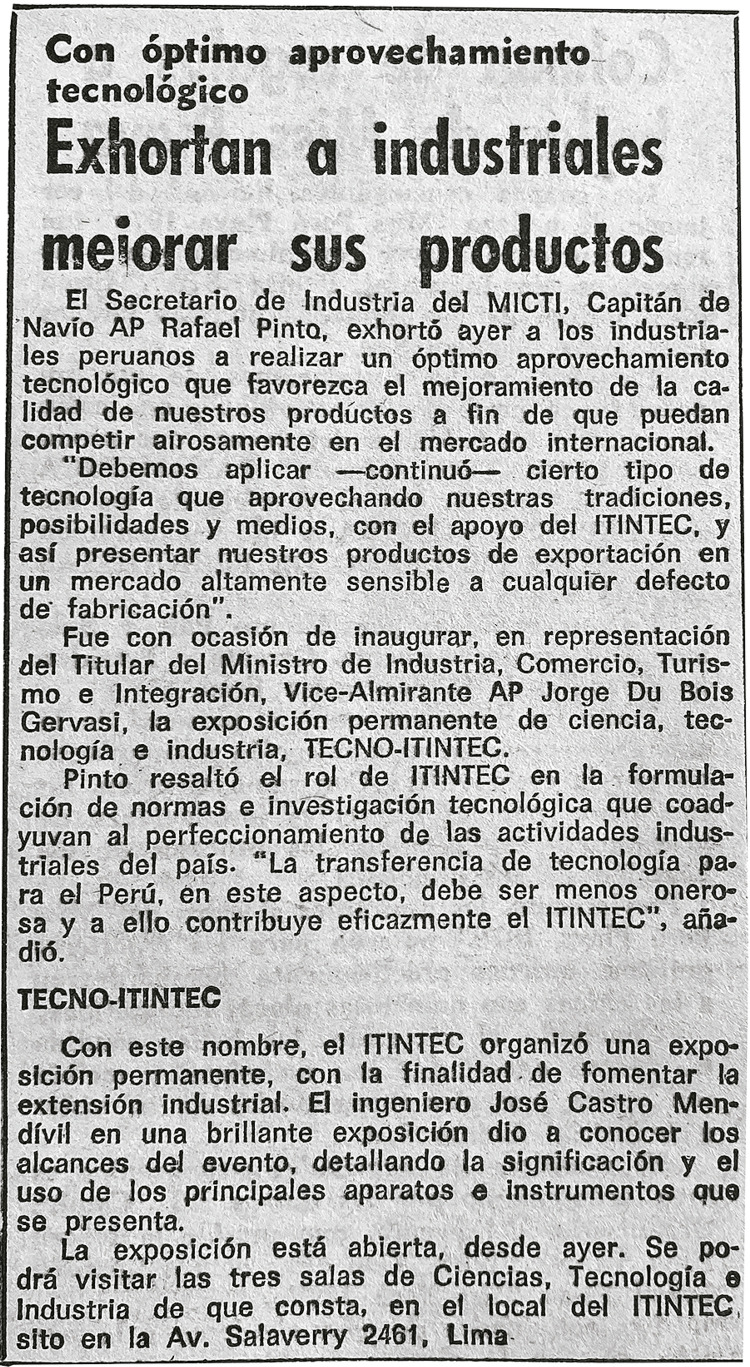
: Story published in the *Expreso* newspaper reporting the inauguration (Source: National Library of Peru)

The press covered the museum’s opening, but the reports focused more on ITINTEC instead of the museum. The article on the inauguration noted the presence of Capitan Rafael Pinto Texeira, who represented the minister of Industry at the event. According to the newspaper, Pinto Texeira said: “We must apply a certain type of technology, taking advantage of our traditions, possibilities, and means, with the support of ITINTEC, to present our export products … the transfer of technology for Peru, in this aspect, should be less onerous, and ITINTEC contributes effectively to this” (Exhortan a industriales mejorar sus productos, 1979). The newspapers did not focus on the museum even when the article purportedly reported on the inauguration.

**Figure 3:**
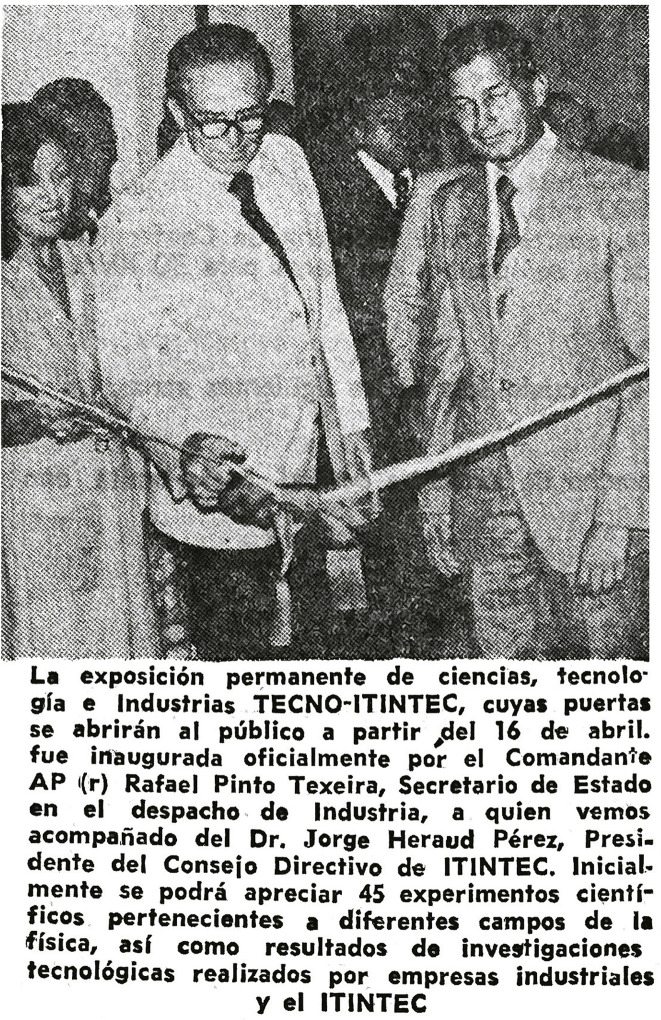
: Reporting on the museum inauguration in *El Comercio* in April 1979 (Source: National Library of Peru)

On April 9, *El Comercio* also featured the museum’s opening in its Industry and Finance section (Aprile, 9 Apr. 1979). The story included a picture of Capitan Pinto Texeira cutting the inaugural ribbon alongside Jorge Heraud and announced the public opening of the institution on April 16 (Figure 3). *El Comercio* republished a short article about the inauguration on April 14 as well as 15, inviting the public to attend and see “forty-five scientific experiments from different fields of physics, as well as the results of technological research carried out by industrial companies and ITINTEC” (Aprile, 15 Apr. 1979).

The official inauguration of TECNO-ITINTEC took place on March 23, 1979, as mentioned in *Expreso* and by Jorge Heraud, with a public opening on April 16 of that same year. Both media appearances corroborated the museum’s opening date, which is crucial for future research on determining whether TECNO-ITINTEC was the first interactive science museum in Latin America.

By the museum opening, Castro Mendívil had been working for months to develop a collection of 45 experiments. He designed the objects to “stimulate each visitor’s interests for a better learning process” (Castro Mendívil, 17 May 1977, p.2). The experiments were interactive and hands-on; visitors interacted with them via buttons, joysticks, films, and other similar techniques intended to convey scientific concepts. Castro Mendívil researched exhibitions from such varied sources as industrial sectors, rural technologies, and children’s entertainment for the design process. To build the experiments, Castro Mendívil had help from some of his students who followed him from PUCP and from his main assistant, Tomas Santillán. Together, they constructed the experiments in a workshop in Castro Mendívil’s house in Miraflores and then incorporated them into the exhibition.

The directors committed to making TECNO-ITINTEC a central institution in the production of science in Lima. The experiments were the museum’s main attraction, along with the popularization activities Castro Mendívil organized. During its first year, TECNO-ITINTEC held 25 conferences, 11 scientific courses, 110 physics talks, and 65 movies provided by European embassies in Lima. From April to December 1979, the museum had 9,869 visitors (7,731 students and 2,138 adults). The number of visitors doubled the following year (Estremadoyro Robles, 13 Jan. 1981).

Castro Mendívil established connections with other museums abroad for inspiration and guidance while building the museum. While he was very skilled in designing the content in the museum, some of the tools and components he needed were unavailable in Peru, and the government restricted imports. He managed to obtain catalogs from companies in the United States that produced scientific equipment for schools and museums, however. He wanted to travel to the United States to obtain these items directly from companies like CENCO, Ealing, and Edmund Scientific. In his reports, Castro Mendívil also mentioned the importance of staying up to date on science museum trends. He received 15 articles that focused on topics including the role of museums in society and their educational purposes. He also wrote to ten museums describing TECNO-ITINTEC’s mission and obtained information from institutions in Chicago, London, and Moscow (Castro Mendívil, 20 June 1977, p.4). The director of Chicago’s Museum of Science and Industry, Victor Danilov, expressed his desire to collaborate and sent information about an association that included 65 science and technology museums around the world. Castro Mendívil also aspired to hire a museum expert from the United States through an organization that arranged collaboration with senior or retired experts from that country called Cuerpo Internacional de Servicios Ejecutivos (known as CISE) (Román de Silgado, 1981).

Through their connections with other museums, Castro Mendívil and Heraud reinforced the need to make ITINTEC part of international museum networks, and these collaboration efforts continued throughout the museum’s history. For example, Heraud recalls visiting science museums whenever he had the opportunity. Later, in 1981, he won an Eisenhower International Fellowship and visited 14 science museums in cities like Boston, Chicago, and Philadelphia as part of his trip to the United States. Although this paper focuses only on the aspects that made the opening of TECNO-ITINTEC possible, it is crucial to understand that Castro Mendívil and Heraud had the idea to establish it as part of the international network of museums. Their efforts included constant communications with other directors and learning about new museology trends to apply at TECNO-ITINTEC.

## More than a personal project

The determination of Jorge Heraud and José Castro Mendívil alone was not sufficient for the museum to be successful: as part of a public institution, it also needed to match the government’s interests in modernization and industrialization. As mentioned, the museum changed its name and functions to align with ITINTEC’s goal of promoting industrial research. While some constraints of the political context affected the museum (such as the inability to import components for the experiments, or limited communication with museums abroad), its founders were able to balance their desire to inspire children’s interest in science with the political and institutional demands of the government and its representatives.

The personal initiative behind the creation of TECNO-ITINTEC did not make the museum an outlier among science museums. Historians have described how some museums were motivated by the personal interests of their founders and promoters. This is the case with institutions such as the Exploratorium and its founder Frank Oppenheimer, or the Harvard Natural Science Museum and professor Louis Agassiz (Andrewes, 2001; Ledbetter, 2013). At the same time, historians have described museums as institutions that support nation-building and governmental projects (Bedini, 1965; Bergers, Van Trijp, 2017; MacDonald, 2002, 1998; Rudolph, 2002; Sheets-Pyenson, 1988; Yanni, 2005). However, personal motivation does not exclude the motivations of nation-building or governmental projects. Museums that result from personal interests are products of their own national and social contexts, and museums that are part of national plans need actors interested in the subject matter to promote and develop them. The TECNO-ITINTEC museum is an example of how its actors balanced their interests and expertise with the government’s expectations for ITINTEC and eventually managed to open the museum.

In the case of ITINTEC, personal motivation and state ideology co-created the museum. The government gave the institute’s directors the autonomy to start a museum that was initially not part of the institute’s functions. The creation of a science popularization project demanded institutional changes inside ITINTEC. The institute’s organization supported industrial research and collaboration with companies and universities, for which they employed engineers and scientists. These actions differed from the tasks associated with starting a science museum, such as planning exhibitions, engaging audiences, and hiring education experts, among the other activities that Castro Mendívil managed.

For the scope of this research, it is not clear how much the museum opening impacted the institute’s daily operations. The museum did not share a location or audience with ITINTEC. In interviews with former interns and workers, they vaguely remembered hearing about the museum, and most had never visited it. The separation between the institutions is reflected in the secondary literature about ITINTEC, where scholars often do not mention the museum. However, both institutions shared the common goal of advancing Peruvian industry and scientific knowledge. Castro Mendívil and Heraud shared the idea that museums serve the purposes of ITINTEC because they “awaken in people an interest and excitement for science, which leads to technological development in our environment and its application to industry” (Castro Mendívil, 22 Mar. 1977, p.2).

The museum was not the only institution at the time dedicated to popularizing science: others were functioning in Lima when it opened, for example the San Marcos Museum of Natural History and the National Museum of Anthropology, as well as the zoo, the planetarium, and the aquarium (Cine astronómico…, 22 Mar. 1979; El Acuario…, 31 Jan. 1976; El Museo..., 2018). There was also another interactive science exhibition called the Dynamic Museum of Science, but the limited information that exists on the topic indicates that it closed shortly before TECNO-ITINTEC. Moreover, Heraud and Castro Mendívil’s motivation at that time was not just to fill a gap in interactive science education, but to offer a different option from those currently available. As Jorge Heraud (7 Nov. 2020) recalls, “people imagined museums as a collection of old objects that allow them to observe the past …, when museums should be extremely alive and interactive.” The museum reports explicitly stated that they did not want to be a historical exhibition like the other museums in Lima.

## The first interactive Peruvian museum

In recent years there have been proposals to reopen a science museum in Peru, from public institutions such as the Peruvian Council of Science and Technology (Consejo Nacional de Ciencia, Tecnología e Innovación Tecnológica, CONCYTEC), the Ministry of Education, the City of Lima, and OEA (Bertone; CONCYTEC, 2019). Most of these projects cited the ITINTEC museum as Latin America’s first interactive science museum, as repeated in sources such as Marticorena, Kuroiwa and Repetto Málaga (2011), Sagasti and Málaga (2018), and Girón (2019).

According to Heraud, they realized ITINTEC was Latin America’s first interactive science museum when comparing ITINTEC’s inauguration date with those of other Latin American museums. He recalled that this happened during a meeting he attended which was sponsored by the United Nations to reopen the ITINTEC museum and attended by representatives of science museums in Buenos Aires, Colombia, and Mexico (Heraud, 7 Nov. 2020). While exploring primary sources for this research, I found the Dynamic Museum of Science, cited above; the first mention of this museum was made in *El Comercio* on January 19, 1976, three years before the opening of TECNO-ITINTEC.

It is debatable whether this initiative was a museum or just a temporary exhibition. According to the newspapers, it was sponsored by the Ministry of Education and located at Jiron Apurimac 550 in Lima. According to José Rivero Herrera (1979, p.66), the Dynamic Museum was part of the educational extension programs created during Velasco Alvarado’s government. It included “scientific experiments for adults and adolescents with a didactic criterion based fundamentally on the participation of the visitor in the execution of the experiment itself.” The museum included experiments that the public could manipulate. Some experiments included “glass blowing techniques, creating microscope lenses, operating and constructing an electric motor, raising guinea pigs” (Con gran éxito…, 25 Jan. 1976; Funciona Museo…, 18 Jan. 1976). The museum was only open in the mornings, and offered guided tours organized by science teachers.

In a report from 1977, Castro Mendívil mentioned visiting the Dynamic Museum and learning “something about their plans and limitations (but) the search has not been deepened much, so as not to create ‘resistance’ to our purpose” (Heraud, 7 Nov. 2020). The ITINTEC directors knew about the dynamic exhibition and suspected that a closer approach could have created friction between the two institutions. However, the TECNO-ITINTEC team did not consider their vision fulfilled by the dynamic exhibition then, and they carried on with their objective to construct their museum.

## Final considerations

The opening of the ITINTEC museum was the result of multiple factors: the military government’s interest in national industry, the economic autonomy of ITINTEC, the personal experiences of Heraud and Castro Mendívil, support from the Board of Directors, and international trends to popularize science. The museum reflected each of these elements in its design, implementation, and operation, making it a unique product of its time and place in Peru.

This essay gathers relevant details about the years prior to the opening of the museum and its first year. Newspaper coverage confirmed that TECNO-ITINTEC was officially inaugurated on March 23, 1979. This date is crucial for future research to determine whether TECNO-ITINTEC was indeed the first interactive science museum in Latin America. Other additional information has been presented, such as its location, opening budget, and communications between the founders and science museums abroad.

The enthusiasm and support of Peruvian scientists would not have been enough without institutional support. ITINTEC’s organization and autonomy from the government made it possible to create the museum, especially because the economic resources were secured through ITINTEC’s funding system even when the museum was not part of ITINTEC’s original charter. Moreover, the museum was in line with the government’s ideas for promoting industry and national development. It is crucial to understand the unique aspects that led to the opening of this museum, since they will be necessary to understand its closure in future research.

The museum was not only shaped by its local context, but also by the external influence of international museums that led to its creation. The Peruvian scientists pushed for new kinds of science popularization by stressing the importance of an interactive museum like the Exploratorium and other museums abroad. With the initial collection of 45 experiments, they invited visitors, especially students, to conduct hands-on experiments, and the founders pushed for a new vision of how to conduct science. The idea of hands-on activity was meant to inspire future scientists in Peru.

This essay constitutes the first effort to gather primary sources related to the opening of TECNO-ITINTEC. Many sources used for this research had not been previously identified by other historians or the actors involved in the museum, such as the reports by Castro Mendívil. Moreover, different archives in Lima hold the documents related to ITINTEC: most are kept in the archives at INDECOPI, the agency of the National Institute for the Defense of Free Competition and the Protection of Intellectual Property, which replaced ITINTEC.

The history of TECNO-ITINTEC remains a crucial time in the contemporary history of science and technology in Peru and Latin America. This research aligns with previous efforts by scholars in the history of science from Latin America which described the role of science museums and science popularization as crucial for the circulation of scientific ideas in the region, along with a predominant role in science education (Falk, Storksdieck, 2005; Marandino, 2005; Mintz, 2005). In this sense, the history of a pioneering science museum is relevant for the history of Peru. Finally, this research represents the initial work to ultimately describe why TECNO-ITINTEC closed in 1993, as well as later failed attempts to reopen in recent decades.

## Data Availability

Articles from newspapers were collected from the National Library of Peru (https://www.gob.pe/bnp). Documents about the ITINTEC can be found in the INDECOPI archive (https://repositorio.indecopi.gob.pe/).
